# A Novel Four Genes of Prognostic Signature for Uveal Melanoma

**DOI:** 10.1155/2022/8281067

**Published:** 2022-04-05

**Authors:** Yan Liu, Huibin Du, Qi Wan, Yan He, Wei Lu, Wenhao Wang, Xiaohui Lv

**Affiliations:** ^1^Department of Ophthalmology, Affiliated Hospital of Weifang Medical University, Weifang City, Shandong Province, China; ^2^Department of Ophthalmology, People's Hospital of Leshan, Leshan City, Sichuan Province, China; ^3^Department of Medical Oncology, Affiliated Hospital of Weifang Medical University, Weifang City, Shandong Province, China

## Abstract

Autophagy and immunity play critical roles in various cancers, but the prognostic impact of autophagy and immunity for uveal melanoma (UM) remains lacking. Therefore, the RNA sequencing of data in the TCGA-UVM dataset was downloaded from UCSC Xena database. The prognostic autophagy- and immunity-related genes (AIRGs) were selected via univariate Cox regression. Next, we applied LASSO method to construct four genes of signature in the TCGA-UVM and verified in another two GEO datasets (GSE84976 and GSE22138). This signature intimately associated with overall survival (OS) time and metastasis-free survival (MFS) time of UM, which could be considered as a prognostic indicator. Besides, by applying risk assessment, the patients of UM can be divided into two subgroups (high/low risk) with different survival time, distinct clinical outcomes, and immune microenvironments. Gene set enrichment analysis (GSEA) manifested that cancer hallmark epithelial-mesenchymal transition and KRAS pathways were positively activated in the high-risk group. Moreover, the high-risk group could be more sensitive to chemotherapies than the low-risk group. Thus, our finding suggested that the four genes of signature closely linked with UM risk and survival can afford more accurate survival prediction and potential therapeutic targets for clinical application.

## 1. Introduction

Uveal melanoma (UM) is the most common primary intraocular malignancy which accounts for 70% of all ocular cancers. It has become a growing global public health concern with about 50% of UM patients dying of metastatic disease [1–3]. Despite there are certain advances in treatment of UM, the prognosis of UM patients is still poor [4, 5]. Thus, it is urgently required to discovery the novel prognostic biomarkers of UM to unveil the underlying potential molecular mechanism as well as therapeutic targets.

Autophagy is an important process mediating intracellular degradation which regulates cellular and biological homeostasis. Deregulation of autophagy has been proven in various pathological and disease processes, including differentiation, development, and tumorigenesis [6]. As for malignant tumors, the function of autophagy in tumorigenesis seems to be a two-edged sword, which may change at different periods [7]. Recently, many researches revealed that autophagy can reduce cell damage and maintain chromosomal stability by eliminating damaged protein and organelles in early phases of cancer progression [8]. In contrast, autophagy seems to promote tumor cell proliferation and escape from immune surveillance at the late stage of cancer [9]. Moreover, autophagy is intimately associated with immune response, inflammation, and therapeutic resistance [10, 11]. For instance, recent researches have showed that therapeutic targets on autophagy will enhance the immune responses and antitumor effects and overcome drug resistances [12, 13]. Thus, it is undoubted that autophagy and immunity play crucial functions in diverse physiological and pathophysiological processes, especially in cancers. In addition, growing researches have demonstrated that autophagy- and immunity-related gene (AIRG) biomarkers are closely correlated with the prognosis of various types of cancer, including glioma, breast cancer, pancreatic ductal adenocarcinoma, and hepatocellular carcinoma [14–16]. In spite of the significant role of AIRGs in various cancers have been proven, the prognostic value for uveal melanoma is poorly understood.

Luckily, large-scale transcriptome datasets are deposited in accessible repositories, such as UCSC Xena website and GEO database, which afforded valuable resources to explore potential signatures in various tumors for different organisms and biological conditions [17].

Therefore, in our research, AIRGs were firstly downloaded from the ImmPort Database website and the Human Autophagy Database. Next, the univariate Cox analysis and LASSO model were performed to construct a robust four genes of signature, which can distinguish different clinical outcomes, immune microenvironment, and chemotherapy response of UM. The present study indicated that the expression of AIRGs takes pivotal roles in the prognosis of UM and could be considered as a prognostic marker for UM therapy.

## 2. Materials and Methods

### 2.1. Gene Expression Profile and Clinical Information

**Figure 1:**
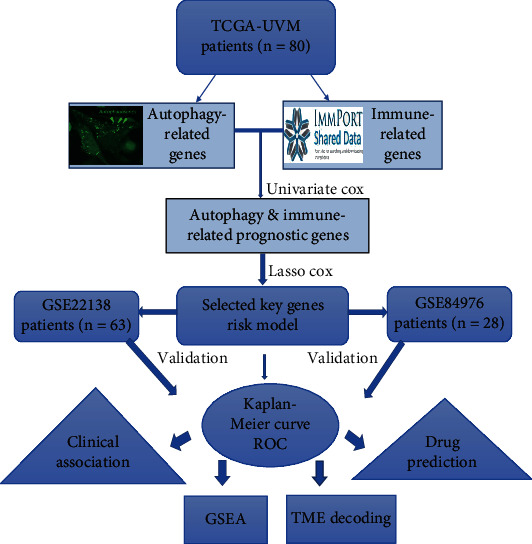
The complete workflow of the analysis in this study.

The RNA sequencing data of 80 UM samples as well as clinical characteristics in the TCGA-UVM dataset were downloaded from UCSC Xena website, which was used for training set. Another two gene expression datasets (GSE84976 and GSE22138) contained 91 UM patients, which was acquired from GEO database and used as outside validation sets. The workflow of overall bioinformatic analysis in this study is shown in Figure 1.

### 2.2. Autophagy- and Immunity-Related Genes (AIRGs)

The autophagy-related genes were collected from the Human Autophagy Database (http://www.autophagy.lu/), which has been demonstrated to take part in the process of autophagy according to the previous studies. Moreover, the list of immunity-related genes was derived from the ImmPort Database (https://immport.niaid.nih.gov), which was the largest accessible human immunology database.

### 2.3. Identification of Prognostic AIRG Model

The overall survival (OS) time was applied to assess the prognostic value of AIRGs in the TCGA-UVM. The univariate cox regression and Benjamini and Hochberg (BH) correcting methods were used to select prognostic AIRGs (adjust *p* values < 0.05 and HR > = 1.10 | HR < = 0.90). Next, we used LASSO algorithm to develop risk model via the prognostic AIRGs. The qualified prognostic AIRGs were selected out to construct the risk system and generate risk scores for UM patients based on the corresponding coefficients. Afterwards, the patients of UM were classified into two subgroups (high or low risk) by the median cutoff value of risk score. The Kaplan-Meier survival curve (log-rank test) was applied to estimate the prognosis of the high-risk and low-risk groups. The sensitivity and specificity of risk model were assessed by receiver operating characteristic (ROC) analysis with area under the curve (AUC).

### 2.4. Associations between Risk Score with Clinical Features and Immune Microenvironment

To evaluate the association between the risk score and clinical features, the risk scores were firstly separated by subgroups of clinical features including age, stage, chromosome 3 status, metastasis, histological type, and vital status. Next, the Kruskal-Wallis or Wilcoxon test was applied to determine significant associations. Moreover, the uni- and multivariable Cox regression were applied to explore the possible prognostic factors of the risk score and clinical features. Besides, several deconvolution methodologies such as CIBERSORT [18], xCell [19], and ESTIMATE [20] were performed to decode tumor microenvironment (TME) contexture. Then, the correlation analysis was conducted to investigate the relationship between risk score and tumor microenvironment.

### 2.5. Gene Set Enrichment Analysis (GSEA)

The GSEA was performed to discover the pathway enrichment and significant molecular mechanisms of the low- vs. high-risk groups by using “clusterProfiler” package [21]. All genes were firstly assessed by “Limma” differential analysis for the low- vs. high-risk groups and then preranked using the log2 fold change of the expression values. Then, the GSEA analysis of the low- vs. high-risk groups in UM was performed; the significant pathways were screen by FDR < 0.05. The cancer hallmarks (h.all.v7.0.symbols) set was used for the GSEA.

### 2.6. Chemotherapeutic Response Prediction

Drug information data was extracted from the Genomics of Drug Sensitivity in Cancer (GDSC) database (https://www.cancerrxgene.org). To explore the likelihood of chemotherapeutic drugs, the “pRRophetic” algorithm was used to predict the chemotherapeutic response for each sample, and the significant chemo drugs were selected by *p* < 0.05 [22].

### 2.7. Statistical Analysis

Every statistical analysis was executed with R software (v.3.5.2) and corresponding statistical packages.

## 3. Results

### 3.1. Prognosis Associated AIRGs

**Figure 2:**
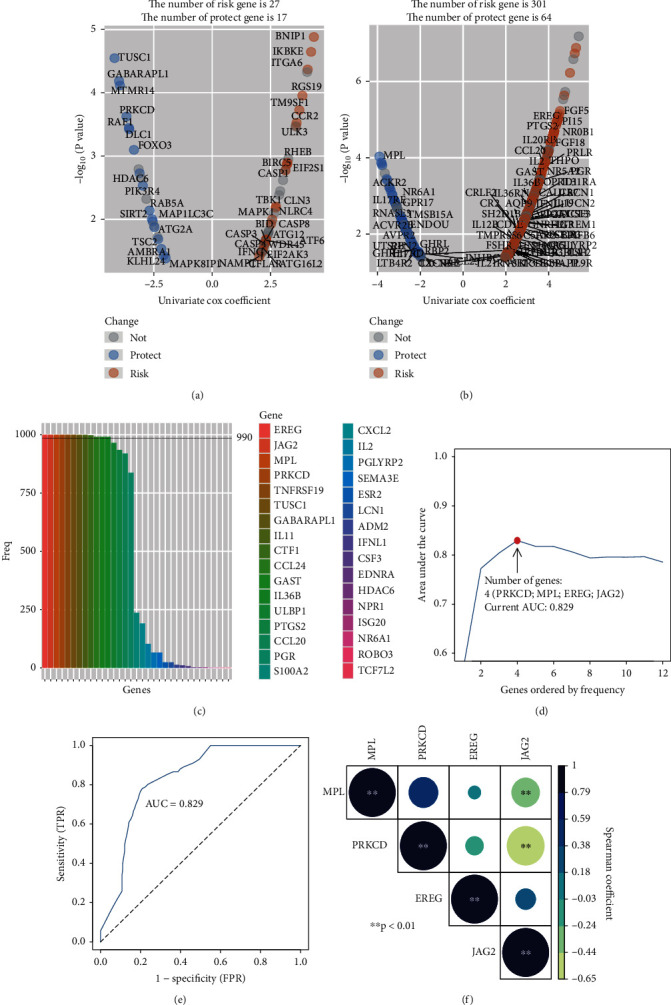
Discovery of autophagy- and immunity-related gene (AIRG) signature for the prognostic prediction. (a) Volcano plot displayed the univariate Cox regression analysis of autophagy-related gens. (b) Volcano plot displayed the univariate Cox regression analysis of immunity-related gens. (c) In 1000 iterations of lasso modeling, there are 33 AIRGs that have been selected for survival prediction but only 12 AIRGs over 990 times to appear in this process. (d) The AUC curve of the AIRG-combined models. The highest AUC value (0.829) at the four genes combination. (e) The 5-year AUC curve of 4 genes of signature (f) Correlation analysis of 4 AIRGs including PRKCD, MPL, EREG, and JAG2. The color spectrum and the size of circle represent the *r* value of the Spearman coefficient. ∗ means *p* < 0.05; ∗∗ means *p* < 0.01.

A number of 222 autophagy-related genes and 1793 immunity-related genes were collected for univariable Cox regression in the TCGA-UVM dataset. According to the selection standard, 44 autophagy-related genes were significantly associated with survival of UM patients, which contained 27 risk genes and 17 protect genes (Figure 2(a)). In addition, 365 immunity-related genes were survival-associated, which consisted of 301 risk genes and 64 genes (Figure 2(b)). Apart from the overlapped genes, a total of 409 AIRGs were identified for the subsequent analysis.

### 3.2. Discovery and Validation of the Four Genes of Prognostic Signature

**Figure 3:**
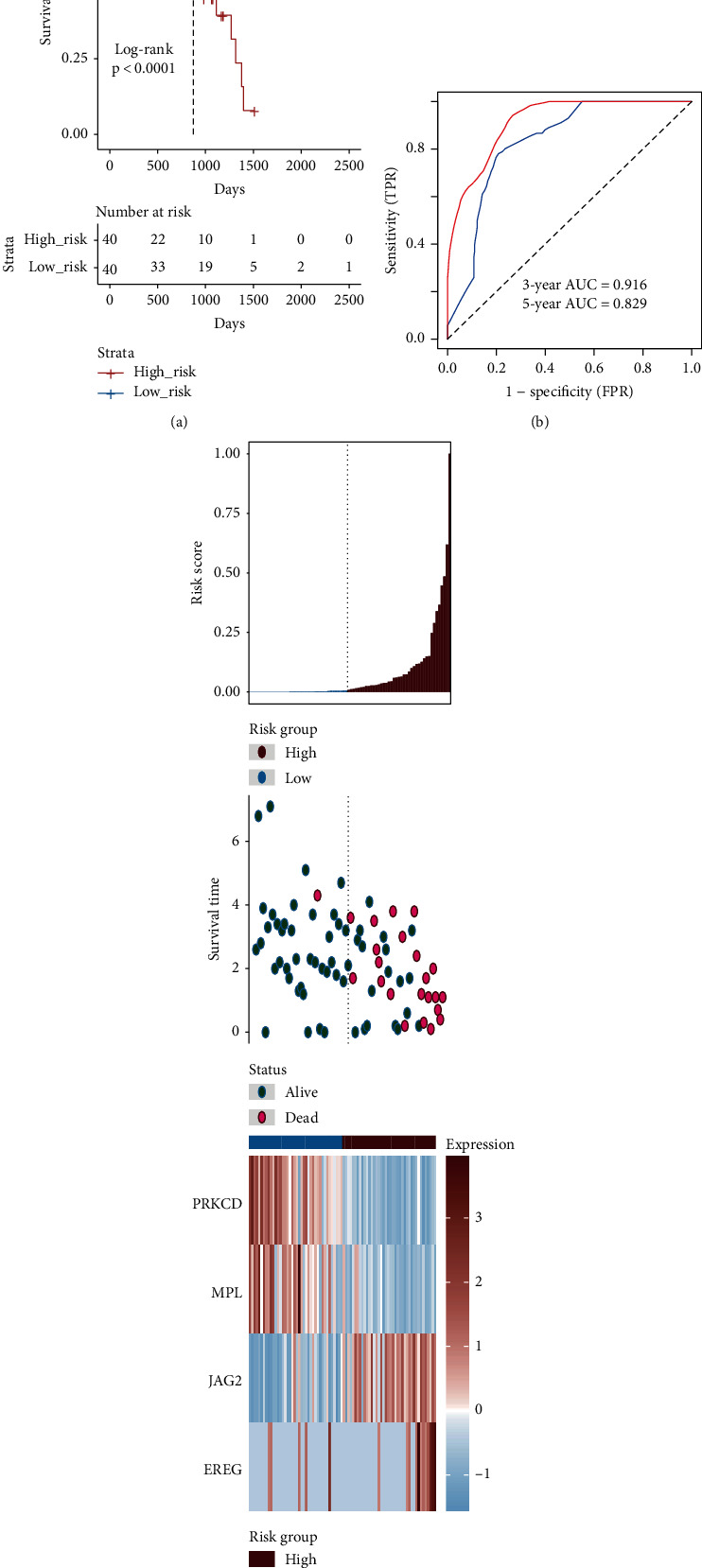
Construction of the 4 genes of signature in the TCGA-UVM to predict the overall survival (OS) of UM patients. (a) The Kaplan-Meier analysis of the high- and low-risk groups in the TCGA-UVM dataset. (b) Receiver operating characteristic (ROC) analysis with area under the curve (AUC) for 4 genes of signature in 3 and 5 years. (c) The risk scores distribution, overall survival time, vital status, and the expression value of 4 genes for 80 UM patients in the TCGA-UVM. From left to right, the bars display risk scores ranked in ascending order.

**Figure 4:**
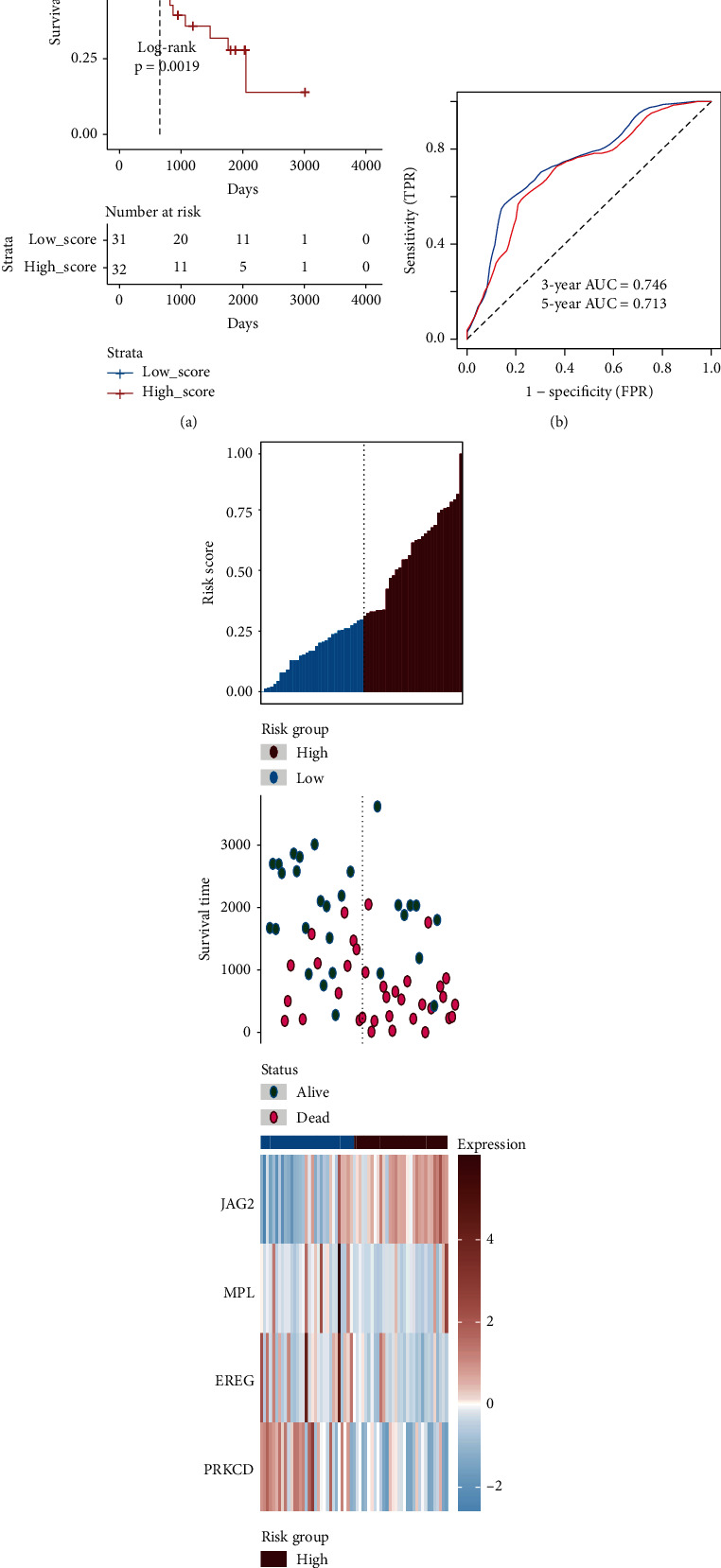
Verification of the 4 genes of signature in the GSE22138. (a) The Kaplan-Meier analysis of the high- and low-risk groups in the GSE22138 dataset. (b) Receiver operating characteristic (ROC) analysis with area under the curve (AUC) for 4 genes of signature in 3 and 5 years. (c) The risk scores distribution, metastasis-free survival, vital status, and the expression value of 4 genes for the 63 UM patients in the GSE22138. From left to right, the bars display risk scores ranked in ascending order.

**Figure 5:**
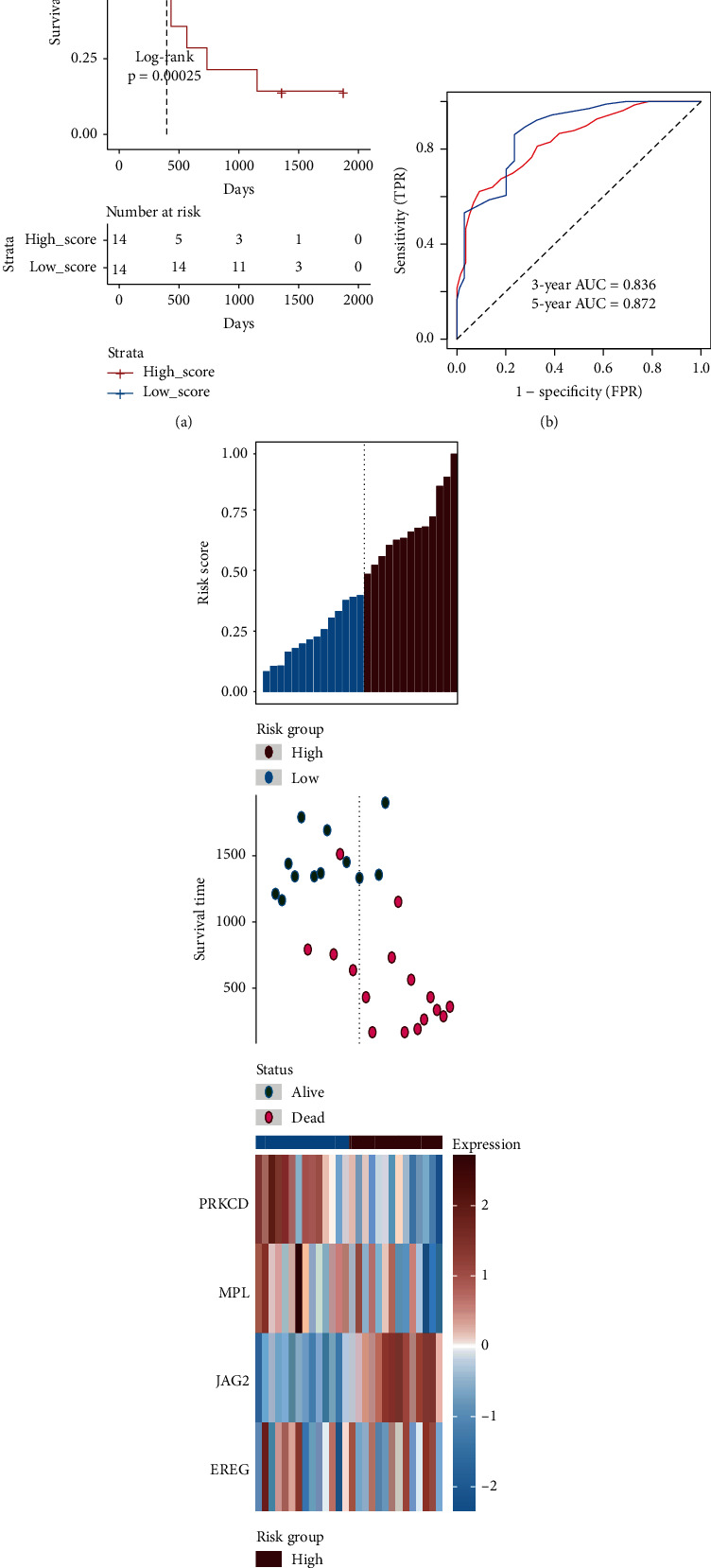
Verification of the 4 genes of signature in the GSE84976. (a) The Kaplan-Meier analysis of the high- and low-risk groups in the GSE84976 dataset. (b) Receiver operating characteristic (ROC) analysis with area under the curve (AUC) for 4 genes of signature in 3 and 5 years. (c) The risk scores distribution, overall survival time, vital status, and the expression value of 4 genes for the 28 UM patients in the GSE84976. From left to right, the bars display risk scores ranked in ascending order.

Firstly, 1,000 iterations of LASSO modeling were performed to evaluate the qualified variables from the 409 AIRGs for constructing prognostic biomarker. Across 1,000 iterations, 12 survival-associated AIRGs were selected out to build biomarker for appearing more than 990 times in LASSO modeling (Figure 2(c)). Moreover, based on the 5-year AUCs of different gene combinations, we found that 4 genes of signature (PRKCD, MPL, EREG, and JAG2) were arrived the max value of 0.829 (Figures 2(d) and 2(e)). The significant correlations were also found among these AIRGs (Figure 2(f)). Finally, a 4-gene signature was identified, and the formula was computed as follows: risk score = ∑_*i*=1_^N^(coef_*i*_ × expr_*i*_). The coef_*i*_ means the Cox coefficient of gene and *N* is the number of gene. The expr_*i*_ represents the relative expression of the gene in risk model. The risk scores of UM patients were calculated by risk formula and next scaled range from 0 to 1. Then, UM patients were divided into high-risk (*n* = 40) and low-risk groups (*n* = 40) by the median cutoff value. The curves of the Kaplan-Meier (KM) survival analysis manifested that high-risk patients have worse prognosis than those in the low-risk with log-rank test *p* value < 0.001 (Figure 3(a)). The ROC curves manifested that the 3- and 5-year AUCs were 0.916 and 0.829, respectively (Figure 3(b)). The distribution risk scores, overall survival, vital status, and corresponding expression of AIRGs in the TCGA-UVM were illustrated in Figure 3(c). To verify the robustness and applicability of signature, the validated analyses were applied in another two GEO datasets (GSE22138 and GSE84976). These datasets were classified into subrisk groups accordingly. The curves of KM revealed the similar results which indicated that the low-risk patients had significantly longer survival time than patients in the high-risk group (GSE22138 log-rank *p* value = 0.0019 and GSE84976 log-rank *p* value < 0.001, respectively) (Figures 4(a) and 5(a)). The 3- and 5-year AUCs in GSE22138 were 0.746 and 0.713 (Figure 4(b)). Higher AUCs (3-year: 0.836 and 5-year: 0.872) were also observed in GSE84976 (Figure 5(b)). The distribution risk scores, metastasis-free survival, vital status, and corresponding expression of AIRGs in GSE22138 were illustrated in Figure 4(c). The distribution of risk scores, overall survival, vital status, and corresponding expression of corresponding AIRGs in GSE84976 were illustrated in Figure 5(c).

### 3.3. Performance Evaluation of Four Genes Signature

**Figure 6:**
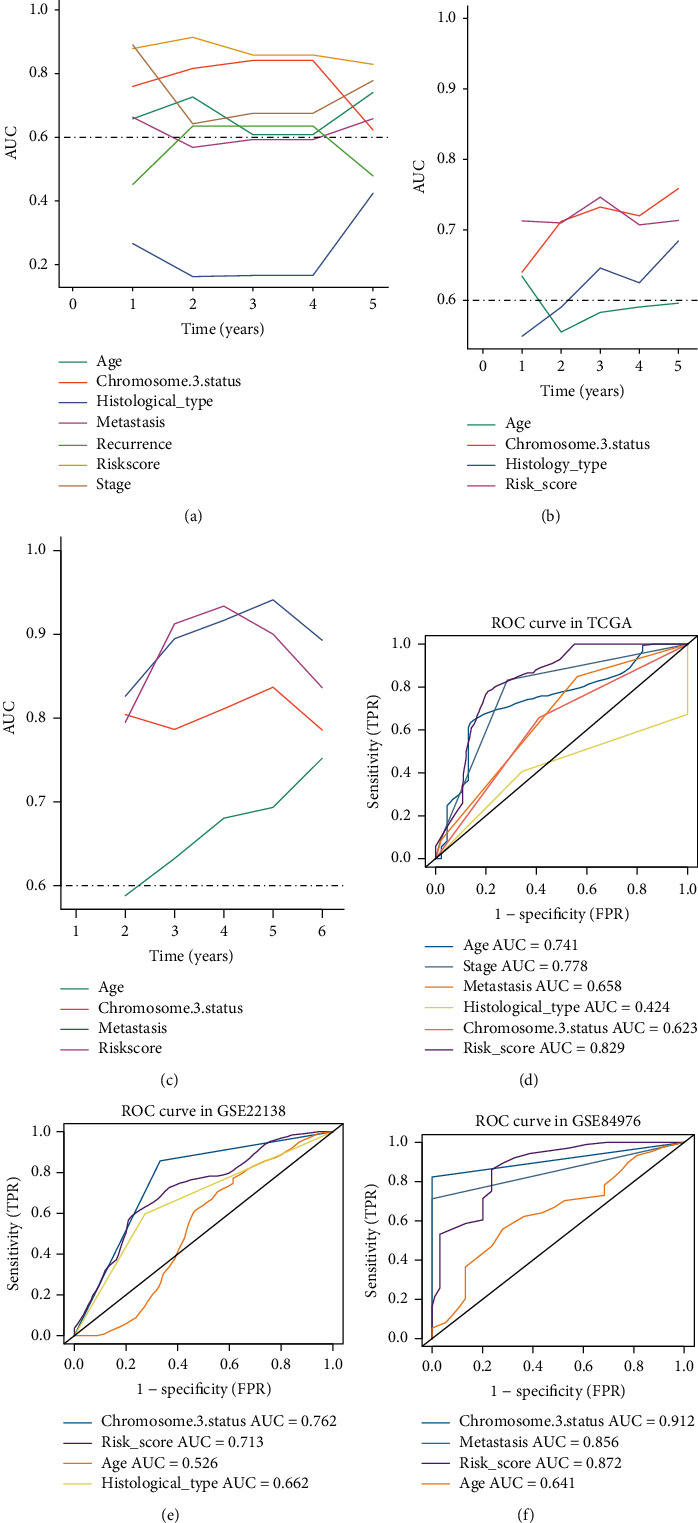
Performance evaluation of the four genes signature. (a) The time-dependent AUC of risk score and clinical features in the TCGA-UVM. (b) The time-dependent area under the curve (AUC) of risk score and clinical features in the GSE22138. (c) The time-dependent AUC of risk score and clinical features in the GSE84976. (d) The 5-year AUCs of age, stage, metastasis, histological type, chromosome 3 status, and risk sore in the TCGA-UVM. (e) The 5-year AUCs of age, histological type, chromosome 3 status, and risk sore in the GSE22138. (f) The 5-year AUCs of age, metastasis, chromosome 3 status, and risk sore in the GSE84976.

To evaluate the performance of four genes signature and other clinical features for prognostic prediction, uni- and multivariable cox regression were conducted by the overall or metastasis-free survival (MFS or OS) time in multiple datasets (Table 1). The results of univariable Cox revealed that stage, age, metastasis, histological type, and risk sore were significantly associated with MFS or OS. According to the multivariate Cox regression analyses, only the risk score was stably remained independent prediction for OS or MFS in the TCGA-UVM dataset (HR = 34.951, 95% CI = 4.891–249.759, *p* < 0.001), GSE22138 dataset (HR = 45.623, 95% CI = 10.025–564.298, *p* < 0.001), and GSE84976 dataset (HR = 3.532, 95% CI = 1.783–9.276, *p* = 0.042). Compared to the time-dependent AUC estimation of traditional clinical variables, the risk score of four genes signature was close to chromosome 3 status and even superior than other variables regardless in the TCGA-UVM (Figure 6(a)), GSE22138 (Figure 6(b)), and GSE84976 (Figure 6(c)). The 5-year AUCs of age, stage, metastasis, histological type, chromosome 3 status, and risk sore in the TCGA-UVM set were 0.741, 0.778, 0.658, 0.424, 0.623, and 0.829, respectively (Figure 6(d)). In GSE22138, the 5-year AUCs of age, histological type, chromosome 3 status, and risk score were 0.526, 0.662, 0.762, and 0.713, respectively (Figure 6(e)). In addition, the 5-year AUCs of age, chromosome 3 status, metastasis, and risk sore in the GSE84976 set were 0.641, 0.912, 0.858, and 0.872, respectively (Figure 6(f)).

### 3.4. Correlation between Signature and Clinical Features

**Figure 7:**
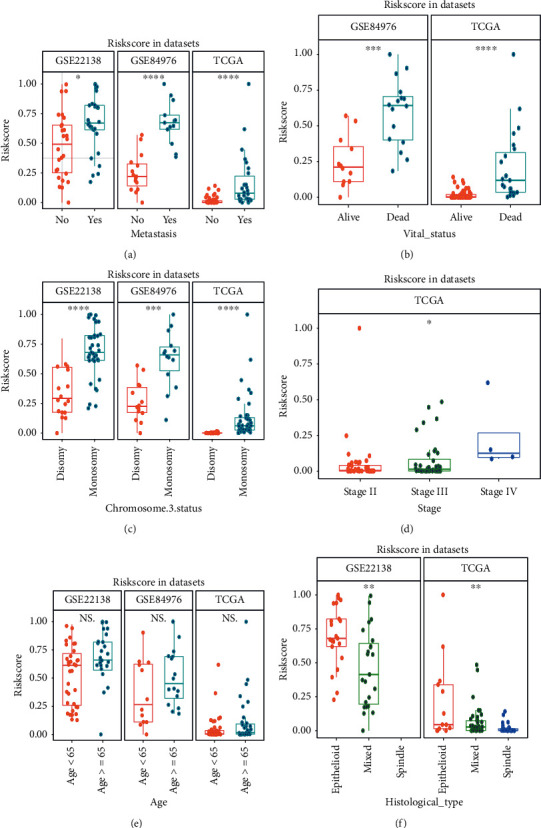
Correlations between risk score of signature and clinical features in multiple datasets. (a) The distribution risk scores for metastasis in the GSE22138, GSE84976, and TCGA-UVM. (b) The risk distribution risk scores for vital status in the GSE84976 and TCGA-UVM. (c) The distribution risk scores for chromosome 3 status in the GSE22138, GSE84976, and TCGA-UVM. (d) The distribution risk scores for tumor stage in the TCGA-UVM. (e) The distribution risk scores for age in GSE22138, GSE84976, and TCGA-UVM. (f) The distribution risk scores for histological type in the GSE22138 and TCGA-UVM. ∗ means *p* < 0.05; ∗∗ means *p* < 0.01; ∗∗∗ means *p* < 0.001; and ∗∗∗∗ means *p* < 0.0001.

The correlation between risk score of signature and clinical features was evaluated, and the results of stratified analyses showed that metastasis (Figure 7(a)), vital status (Figure 7(b)), chromosome 3 status (Figure 7(c)), tumor stage (Figure 7(d)), and histological type (Figure 7(e)) were significantly correlated with the risk score. Other clinical feature, such as age (Figure 7(f)), has no relationships with the risk score. Thus, the signature was intimately correlated with these traditional clinical features.

### 3.5. Associations between Risk Score and Immune Microenvironment

**Figure 8:**
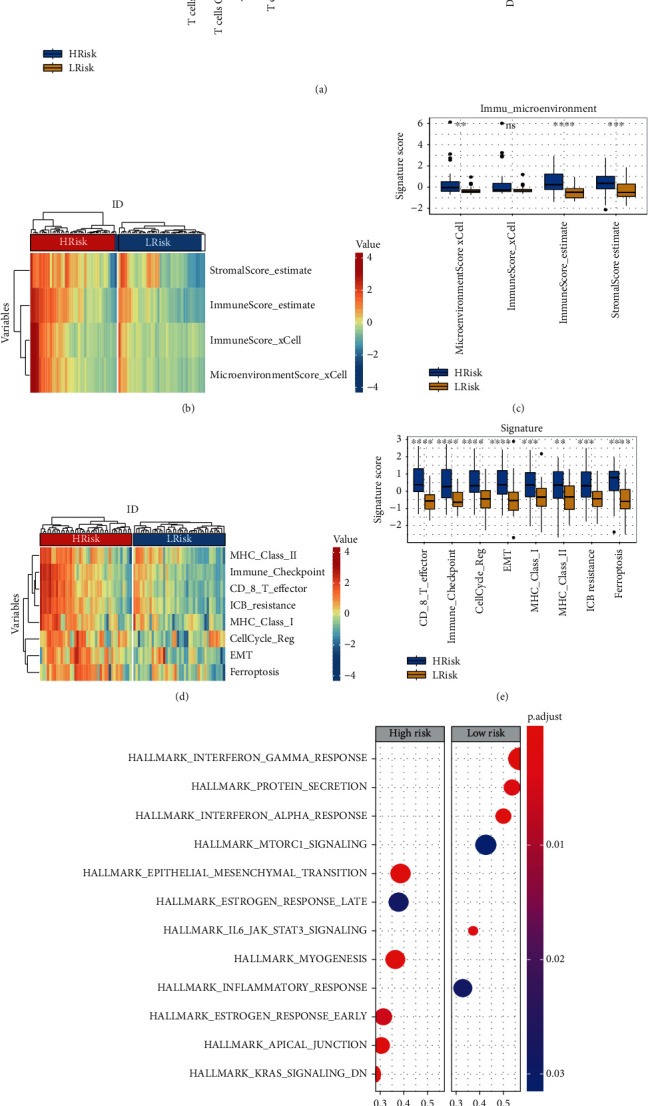
Associations between risk score of signature with immune microenvironment and cancer hallmark. (a) The subgroup analysis of 22 immune infiltrating cells between the high- and low-risk groups. (b) Heat map of microenvironment score, immune score, and stromal scores between the high- and low-risk groups. Red color indicates higher score, and blue color refers to lower score. (c) Box plots of microenvironment scores, immune score, and stromal scores between the high- and low-risk groups. (d) Heat map of scores for immunity- and tumor-related pathway between the high- and low-risk groups. Red color indicates higher score, and blue color refers to lower score. (e) Box plots of scores for immunity- and tumor-related pathway between the high- and low-risk groups. ∗ means *p* < 0.05; ∗∗ means *p* < 0.01; ∗∗∗ means *p* < 0.001; and ∗∗∗∗ means *p* < 0.0001. (f) GSEA analysis of high- vs. low-risk score groups.

Through the CIBERSORT algorithm, 22 kinds of immunity cells were selected for the stratifying analysis. The box plots showed that the fractions of T cell CD8, T cells CD4, T cells regulatory, NK cells activated, monocytes, macrophages M1, and mast cells in the high-risk subgroup significantly different from those in the low-risk group (Figure 8(a)). Next, the microenvironment score, immune score, and stromal score were calculated by applying xCell and ESTIMATE algorithms. The heat map of microenvironment score, immune score, and stromal scores between high- and low-risk groups was illustrated in Figure 8(b). The box plots suggested that the high-risk group has a higher microenvironment, immune score, and stromal score than the low-risk group (Figure 8(c)). Moreover, some important immunity-related pathways (such as MHC class-II, MHC class-I, immune checkpoint, CD8 T effector, and ICB resistance) and tumor-associated pathways (like cell cycle, EMT, and ferroptosis) were assessed by single sample GSEA (ssGSEA) algorithm [23]. The heat map (Figure 8(d)) and subgroup (Figure 8(e)) analyses revealed that the scores of these pathways in the high-risk subtype were generally higher than those in the low-risk subtype.

### 3.6. GSEA

According to selected criterion, we confirmed that six positive correlated pathways were enriched in the high-risk group, including epithelial-mesenchymal transition (EMT), estrogen response late, myogenesis, estrogen response early, apical junction, and KRAS signaling. Moreover, six cancer hallmark pathways contained interferon gamma response, protein secretion, interferon alpha response, MTORC1 signaling, JAK STAT3 signaling, and inflammatory response actively associated with the low-risk group (Figure 8(f)).

### 3.7. High-Risk Subgroup More Sensitive to Chemo Drugs

**Figure 9:**
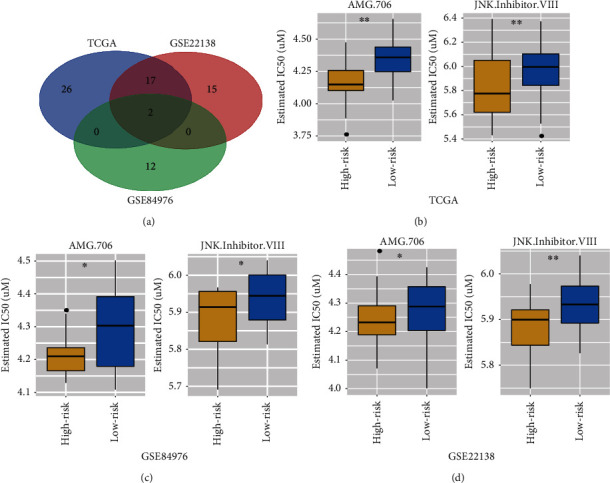
Differential response of drugs. (a) The Venn plot of the three datasets including TCGA-UVM, GSE84976, and GSE22138. 19 kinds of compounds are shared in the TCGA-UVM and GSE22138. Only 2 kinds of compounds (AMG.706 and JNK.Inhibitor.VIII) are shared in the TCGA-UVM, GSE84976, and GSE22138. (b) The box plots for the estimation of IC50 in the AMG.706 and JNK.Inhibitor.VIII at the TCGA-UVM. (c) The box plots for the estimation of IC50 in the AMG.706 and JNK.Inhibitor.VIII at the GSE84976. (d) The box plots for the estimation of IC50 in the AMG.706 and JNK.Inhibitor.VIII at the GSE22138. ∗ means *p* < 0.05 and ∗∗ means *p* < 0.01.

Due to the resistance for regular chemotherapeutics, scientists are struggling to discover new potential compounds for UM. Therefore, the GDSC database was used to predict sensitivity of chemo drugs. Based on the selecting criteria, 45 kinds of drugs were screened out in the TCGA-UVM set. 34 and 14 kinds of drugs were identified in the GSE22138 and GSE84976, respectively (Table 2). The Venn plot analysis suggested that AMG.706 and JNK.Inhibitor.VIII were the common drugs among the three datasets (Figure 9(a)). We observed significant differences for the estimation of IC50 between the high- and low-risk group, and these drugs in the high-risk group could be more sensitive to chemotherapies (Figures 9(b)–9(d)).

## 4. Discussion

Like many kinds of tumor, the prognosis of uveal melanoma is largely dependent on the early diagnosis and treatment [3]. With the growing clinical application of new therapeutic targets, the present studies are mainly focused on the detection of novel prognostic biomarkers which are often used to assess disease risk and early diagnosis [24–27]. Clinical application of prognostic biomarkers can early guide high-risk patients who undergone individual treatment and management, even prevent life-threatening metastases [28–32]. For example, massive previous researches were struggled to identified potential genes, miRNAs, or DNA methylations combination biomarkers via bioinformatics to predict survival of UM [33–35]. Hence, as far as we known, our study is the first time to investigate the prognostic role of AIRG biomarker in UM by applying multiple public datasets. Then, we constructed a robust 4 genes of signature in UM prognosis and validated in multiple independent datasets. Our prognostic biomarker can subsequently classify patients into subgroups with different survival events. Compared to traditional clinical features, our AIRG biomarker achieved higher accuracy than most of clinical indicators (e.g., stage, age, metastasis, and histology type). Notably, the results of multivariate cox regression manifested that the risk score of 4-gene signature can afford a robustly accurate prediction of OS or MFS in UM and could be considered as an independent prognostic model.

In this research, we developed a prognostic biomarker with four selected AIRGs (PRKCD, MPL, EREG, and JAG2), which classified the UM patients into high- and low-risk groups. The Kaplan-Meier curves revealed that patients in the high-risk group have a poor survival. Among the four AIRGs, almost all of the genes have been reported to be associated with prognosis in other malignancies. For instance, PRKCD (protein kinase C delta) is a member of protein kinase C (PKC) family which is a critical regulator of the chemosensitivity in cancers such as non-small-cell lung cancer, ovarian cancer, and prostate cancer [36–38]. Additionally, PRKCD has been regarded as a novel prognostic biomarker for ovarian cancer patient response to overall disease-specific survival [39]. MPL (thrombopoietin receptor) is a member of homodimeric class I receptor family, which mainly exerted its influence on regulation of megakaryopoiesis, formation of immune synapses, and enhancing antitumor function [40, 41]. Nishimura et al. recently reported that C-MPL can work as a novel immunotherapeutic target to promote antitumor activity of T cells by mediation of cytokine pathways [42]. Therefore, MPL could be considered as a potential therapeutic target for UM patients in the future. EREG (epiregulin) belongs to the epidermal growth factor family and benefits to wound healing, tissue repair, and inflammation in normal physiology, while dysfunction of epiregulin will increase the progression of different malignancies. Previous studies revealed that the overexpressed EREG closely correlates with many cancers, including prostate cancer, colon cancer, breast cancer, and bladder cancer [43–46]. What is more, Asnaghi et al. reported that JAG2 plays an important role in promoting tumor cells growth and metastasis in uveal melanoma, which could serve as a new therapeutic target to further investigation [47].

To better understand the underlying molecular functions in the subtypes of UM, the GSEA analyses were conducted to find the potential 4 genes of signature-related pathways in cancer hallmarks database. Cancer hallmark pathways such as epithelial-mesenchymal transition (EMT) and KRAS signaling were positively enriched in the high-risk expression subgroup. It is generally recognized that EMT pathway is a crucial factor to control the clinical outcome of patients with various tumors. Asnaghi et al. proven that EMT-related factors will promote invasive properties of uveal melanoma cell and finally lead to poor prognosis [48]. Besides, Kirsten rat sarcoma viral oncogene homolog (KRAS) regard as the most lethal cancer-related proteins takes a crucial role in human aggressive cancers [23]. Previous study demonstrated that the activation of KRAS-ERK signaling can promote development and migration of uveal melanoma cells [49]. Meanwhile, we observed that interferon gamma, interferon alpha, and inflammatory response were actively enriched in the low-risk subgroup. It is widely acknowledged that interferon not only has antiviral activities but also powerful antitumor activities via various inflammatory response pathways [50]. Substantial evidences suggested that interferon alpha and gamma can inhibit the proliferation of uveal melanoma cells in vitro and indicated a potential adjuvant treatment for patients with uveal melanoma [51, 52]. Therefore, we can reasonably believe that our results were consistent with the previous conclusions indicated that the high-risk subgroup has a poor survival in UM due to the positive enrichment of EMT and KRAS as well as the negative association with interferon gamma and interferon alpha.

By using various TME-decoding algorithms, we firstly observed that the high-risk group has a significant higher microenvironment score, immune score, and stromal score than the low-risk group, which indicated that a distinct TME contexture exists between the two subtypes. Next, via CIBERSORT assessment, we found that the infiltration of CD8+ T cells and M1 macrophages were highly expressed in the high-risk group. What is more, immunity-related pathways including MHC class-II, MHC class-I, immune checkpoint, CD8 T effector, ICB resistance, and tumor-associated pathways containing cell cycle, EMT, and ferroptosis were all positively enriched in the high-risk group. These observations appear counterintuitive because high infiltration of immune cells and activation of immune-related pathways are usually correlated with good prognosis in many solid tumors [53]. However, the increasing researches revealed that an immunosuppressive environment definitely exists in UM. For eye as an immune-privileged organ, the high infiltration of immune cells and activation of immune immune-related pathways will promote ocular tumor immune evasion. Notably, recent studies also demonstrated that the increased level of CD8+ T cells is a predictive factor for poor prognosis in UM, and the accumulation of macrophages is poorly associated with the prognosis of melanoma patients [54–56]. In addition, Valdor and Macian reviewed the articles and proposed that the activation of autophagy could lead to strong MHC class antigen presentation and increased CD8+ T cell activity [57]. Therefore, it is logical to speculate that our signature could become an effective therapy target in UM due to the correlation with autophagy-regulated immune response.

Furthermore, in order to explore the different chemotherapeutic response between the high- and low-risk subgroups, GDSC drug database was performed to explore the candidate drugs, and the results showed that the high-risk subgroup is more sensitive to chemo drugs. Moreover, the three datasets showed that the AMG.706 and JNK.Inhibitor.VIII were the commonly enriched drugs. The estimated IC50 of the AMG.706 and JNK.Inhibitor.VIII in the high-risk group is significantly lower than the low-risk groups. The AMG.706 (motesanib) is a multikinase inhibitor which significantly inhibits VEGF-induced vascular permeability and angiogenesis and induces regression in tumor xenografts [58]. The Rosen et al. study showed that the AMG.706 has favorable pharmacokinetics and good tolerability in advanced refractory solid tumors [59]. A phase III clinical trial suggested that the AMG.706 can significantly improve progression-free survival of patients with stage IV lung cancer [60]. The JNK.Inhibitor.VIII, that is, a JNK inhibitor, can inhibit the cell apoptosis and block the phosphorylation of c-Jun and JNKAR1 which promote tumorigenesis and metastasis of cancers [61]. Emerging evidences proven that the JNK inhibitors could be regarded as potential targeting therapy in various type of tumors [62]. Hence, it is reasonable to believe that the AMG.706 and JNK.Inhibitor.VIII could be considered as potential chemo drugs for further clinical treatment of UM.

To sum up, our research constructed a robust 4 genes of signature prognostic biomarker in UM which could be regard as an independent prognostic model in clinical application. The patients with the high risk score could benefit more chemotherapy. However, the discoveries are obtained from the bioinformatics analysis and needed to validate in further basic and clinical studies.

## Figures and Tables

**Table 1:** Uni- and multivariable Cox regression of clinicopathologic feature associated with overall or metastasis-free survival in the TCGA-UVM, GSE84976, and GSE22138 datasets. ∗ means *p* < 0.05 and ∗∗ means *p* < 0.01.

	Univariate analysis				Multivariate analysis			
TCGA (*n* = 80)								
Marker	unicox_p	HR	Lower .95	Upper .95	mutlicox_p	HR	Lower .95	Upper .95
Age	0.020∗	1.058	1.009	1.109	0.056	1.055	0.999	1.115
Gender	0.354	1.747	0.537	5.686				
Stage	0.004∗∗	8.557	1.998	36.653	0.906	0.902	0.163	4.990
M	0.024∗	7.359	1.302	41.579	0.019∗	16.395	1.599	168.124
N	0.086	0.364	0.115	1.155	0.001∗∗	0.024	0.003	0.198
T	0.239	1.831	0.668	5.013				
Histological_type	0.017∗	0.123	0.022	0.688	0.194	0.224	0.023	2.145
Recurrence	0.257	4.018	0.363	44.528				
Chromosome 3 status	0.997	711.563	28.884	11842.042				
Risk score	0.000∗∗	57.610	11.103	298.911	0.000∗∗	34.951	4.891	249.759
GSE84976 (*n* = 28)								
Age	0.142	1.030	0.990	1.071				
Chromosome 3 status	0.001∗∗	9.444	2.636	33.832	0.821	1.253	0.179	8.783
Metastasis	0.000∗∗	28.513	5.938	136.924	0.008∗∗	20.930	2.249	194.820
Risks core	0.000∗∗	99.53	9.230	1073.373	0.000∗∗	45.623	10.025	564.298
GSE22138 (*n* = 63)								
Age	0.113	1.021	0.995	1.049				
Gender	0.316	0.702	0.352	1.400				
Chromosome 3 status	0.001∗∗	5.990	2.061	17.406	0.159	3.803	0.591	24.474
Histological_type	0.065	2.123	0.954	4.724				
Risk score	0.003∗∗	5.340	1.313	10.532	0.042∗	3.532	1.783	9.276

**Table 2:** The prediction of chemotherapeutic response for each datasets. The significant chemo drugs and *p* values were listed.

TCGA				GSE22138				GSE84976	
Drug	*p* value	Drug	*p* value	Drug	*p* value	Drug	p value	Drug	*p* value
GW.441756	0.000	Temsirolimus	0.000	AZD.0530	0.000	NVP.TAE684	0.000	X681640	0.003
ABT.888	0.000	SB.216763	0.000	A.770041	0.000	Temsirolimus	0.000	GSK.650394	0.004
BMS.509744	0.000	PF.4708671	0.000	GDC0941	0.000	MK.2206	0.000	Vorinostat	0.007
AZD6482	0.000	PD.173074	0.000	JNK.Inhibitor.VIII	0.000	WZ.1.84	0.002	Epothilone B	0.009
BMS.536924	0.000	Lenalidomide	0.000	BMS.536924	0.001	PF.02341066	0.002	PF.562271	0.011
AZD.2281	0.000	Methotrexate	0.000	AP.24534	0.001	Methotrexate	0.002	ZM.447439	0.018
GDC.0449	0.000	RO.3306	0.000	BMS.509744	0.002	PD.173074	0.003	Doxorubicin	0.020
Lapatinib	0.000	VX.680	0.000	Gefitinib	0.004	WH.4.023	0.003	JNK.Inhibitor.VIII	0.021
KU.55933	0.000	Nilotinib	0.000	ATRA	0.004	SB.216763	0.004	Gemcitabine	0.022
JNK.Inhibitor.VIII	0.001	PLX4720	0.001	Dasatinib	0.005	Lapatinib	0.006	Bleomycin	0.028
AICAR	0.001	X17.AAG	0.001	Imatinib	0.007	Nilotinib	0.007	Etoposide	0.035
AMG.706	0.001	Metformin	0.001	Erlotinib	0.008	KU.55933	0.018	AMG.706	0.041
BMS.708163	0.003	SL.0101.1	0.002	AZD6482	0.016	WO2009093972	0.032	BI.D1870	0.042
Gefitinib	0.004	MK.2206	0.003	GW.441756	0.018	Z.LLNle.CHO	0.036	Thapsigargin	0.042
BMS.754807	0.004	S.Trityl.L.cysteine	0.003	Elesclomol	0.031	PLX4720	0.040		
GW843682X	0.005	VX.702	0.007	GNF.2	0.036	Pazopanib	0.046		
EHT.1864	0.006	OSI.906	0.007	CGP.60474	0.040				
BI.2536	0.007	PF.02341066	0.011	AMG.706	0.041				
A.443654	0.016	Mitomycin C	0.014						
Cyclopamine	0.019	Sorafenib	0.015						
A.770041	0.027	Nutlin.3a	0.027						
CGP.60474	0.043	MS.275	0.034						
AZ628	0.049								

## Data Availability

The datasets generated for this study can be found in the GEO database (GSE22138 and GSE84976; https://www.ncbi.nlm.nih.gov/geo/) and UCSC Xena website (TCGA-UVM; https://gdc. http://xenahubs.net).
